# Newborn with Gastroschisis associated with Limb Anomalies

**DOI:** 10.21699/jns.v6i1.443

**Published:** 2017-01-01

**Authors:** Phyu Phyu Win

**Affiliations:** Paediatric Department, Faculty of Medicine and Health Sciences, UCSI University, Malaysia

**Keywords:** Newborn, Gastroschisis, Limb anomalies, Maternal age

## Abstract

Gastroschisis is often found together with other extra intestinal conditions such as limb, spine, cardiac, central nervous system and genitourinary abnormalities. There are reports of its association with young maternal age. The cases presented here highlight the association of gastroschisis with limbs anomalies and young maternal age.

## INTRODUCTION

In most cases, gastroschisis is an isolated defect.[1] In contrast, omphalocele is more likely to be associated with other major genetic or somatic malformations. In spite of widespread acceptance of a low rate of concurrent malformations with gastroschisis, a careful review on published data revealed the rates of malformation association with gastroschisis ranging from 5% to 27%.[2] We report two newborns with concurrent presence of gastroschisis and limbs anomalies.

## CASE SERIES

**Case 1:**

A premature (35-weeks) baby girl was delivered by normal spontaneous vaginal delivery at a district hospital in Irrawaddy division, Myanmar in 2010. Her mother was a 22-year old primigravida with uneventful antenatal history. She had attended regular antenatal visits at a rural health center where ultrasound scanning was not available. She was non-smoker, non-alcoholic and had no history of medical illness. She did not take any medication except haematinics during the pregnancy. She was a housewife and had no history of exposure to radiation. Her husband was a 24-year-old farmer and was healthy. The baby was delivered with good APGAR score, weighing 2.4 kg. The liquor was meconium-stained. There were intestinal coils protruded from the umbilicus. Fixed flexion deformity of hips, hyperextended knees and talipes equinovarus deformity of right foot were seen. Both elbows had fixed flexion deformity and the wrist joints were hyper extensible. The number of digits was normal (Fig. 1) Spine was normal and anus was patent. There was no cardiac murmur. After resuscitative measures, she was transferred to a tertiary hospital. She died at 96-hours of life. Surgery was not done as the condition was not stable.

**Figure:**
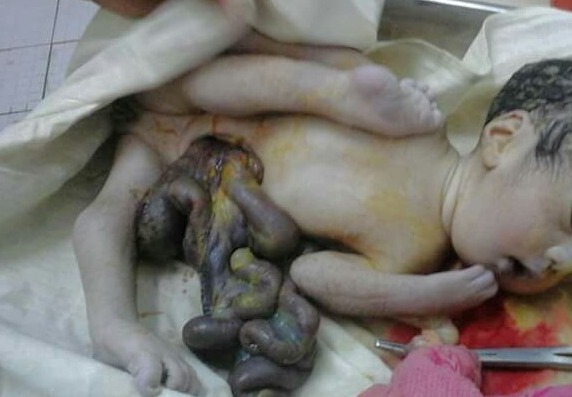
Figure 1. Photograph of newborn showing herniation of small and large intestine through a right paraumbilical wall defect with associated deformed limbs (Case 1).

**Case 2:**

A 17-year old primigravida lady delivered a newborn premature (36-weeks) baby girl via emergency LSCS due to poor progress of the labour at a district hospital in Malaysia in 2013. Her antenatal history was uneventful and there was no history of any risk factors. Antenatal ultrasound scan revealed no abnormality. The baby was born with good APGAR score, birth weight of 2.5 kg and there was evisceration of huge, dilated and twisted intestinal coils without any covering sac. The hips and knees were flexed and twisted; bilateral talipes equinovarus deformity was seen. The muscles around the joints were thin and weak. Wrist joints were hyper extensible. The number of digits in both upper limbs and lower limbs were normal (Fig.2) She was subsequently transferred to the tertiary center for further surgical management. Operative findings revealed atresia at ileum, measuring 2 feet (60 cm) in length, with some necrotic areas. The atretic segment was resected, and reduction of the dilated bowel and end-to-end anastomosis were performed. The post-operative period was uneventful. She was discharged home at six week of life. During the stay at the tertiary hospital, echocardiogram was done and it was found normal. At 2 months of age, she was brought to the district hospital with 3-day history of vomiting and abdominal distension. She was very ill and lethargic. The skin was mottled and perfusion was poor. Despite immediate resuscitative measures, she died within 30 minutes of arrival to the hospital.

**Figure:**
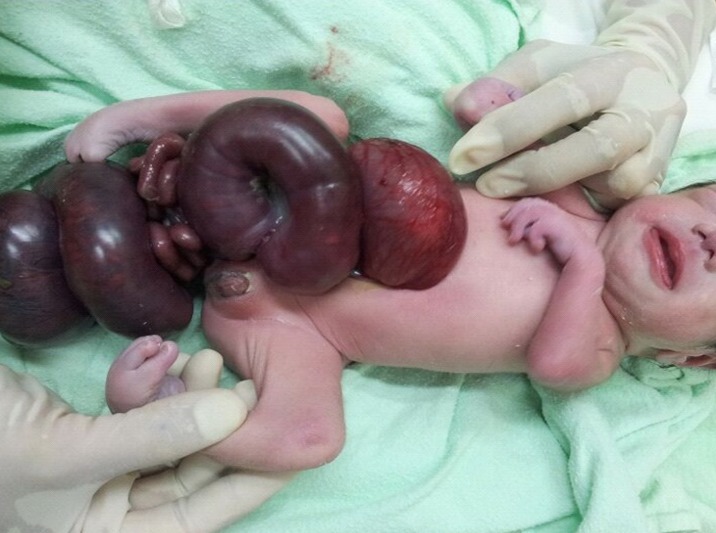
Figure 2. Photograph of newborn showing herniation of twisted and dilated coils of intestines through a right paraumbilical wall defect with associated deformed limbs (Case 2).

## DISCUSSION

Gastroschisis may be associated with gastrointestinal anomalies such as intestinal stenosis, atresia and malrotation, and extra intestinal conditions such as limb, spine, cardiac, central nervous system and genitourinary abnormalities. In the cases presented above, both babies have gastroschisis associated with limbs anomalies.

Benjamin and Wilson [1] retrieved data from The Texas Birth Defect Registry (TBDR) from 1999 to 2008, on anomalies associated with gastroschisis and omphalocele, found out overall prevalence of gastroschisis was 4.8 per 10,000 births and rate of associated anomalies with gastroschisis was 32%, including musculoskeletal anomalies which contributed 9.6%. Musculoskeletal abnormalities reported include limb reduction defects, clubfoot and skeletal dysplasia.[2] Mastroiacovo et al [3] found a 2.2% incidence of associated limb anomalies with gastroschisis.

In our case both mothers were young and primigravida. Tan et al [4] reported median maternal age of 21.0 years (interquartile range 19–25 years) after surveying on 539 cases of gastroschisis in England and Wales. Penman et al [5] reported a twelve-fold increased risk for gastroschisis in women less than 20 years of age at the time of conception compared with older women, suggesting a link between young maternal age and prenatal vascular disruptions, but the evidence for this is unconvincing.

The long-term outcome for omphalocele depends on its associated anomalies, whereas the babies with gastroschisis usually achieve normal growth and development as they progress through childhood. The limb contractures do not need immediate intervention and are not fatal.

## Footnotes

**Source of Support:** Nil

**Conflict of Interest:** None declared
